# A Robust and Integrated Visual Odometry Framework Exploiting the Optical Flow and Feature Point Method

**DOI:** 10.3390/s23208655

**Published:** 2023-10-23

**Authors:** Haiyang Qiu, Xu Zhang, Hui Wang, Dan Xiang, Mingming Xiao, Zhiyu Zhu, Lei Wang

**Affiliations:** 1 School of Naval Architecture and Ocean Engineering, Guangzhou Maritime University, Guangzhou 510725, China; heu_wanghui@126.com (H.W.); xiangdan2000@163.com (D.X.); xmingm@gzmtu.edu.cn (M.X.); 2 School of Automation, Jiangsu University of Science and Technology, Zhenjiang 212013, China; 211210301424@stu.just.edu.cn (X.Z.); zzydzz@163.com (Z.Z.); 3 State Key Laboratory of Information Engineering in Surveying, Mapping and Remote Sensing, Wuhan University, Wuhan 430072, China; lei.wang@whu.edu.cn

**Keywords:** visual odometry, optical flow tracking, feature point method, ORB_SLAM3

## Abstract

In this paper, we propose a robust and integrated visual odometry framework exploiting the optical flow and feature point method that achieves faster pose estimate and considerable accuracy and robustness during the odometry process. Our method utilizes optical flow tracking to accelerate the feature point matching process. In the odometry, two visual odometry methods are used: global feature point method and local feature point method. When there is good optical flow tracking and enough key points optical flow tracking matching is successful, the local feature point method utilizes prior information from the optical flow to estimate relative pose transformation information. In cases where there is poor optical flow tracking and only a small number of key points successfully match, the feature point method with a filtering mechanism is used for posing estimation. By coupling and correlating the two aforementioned methods, this visual odometry greatly accelerates the computation time for relative pose estimation. It reduces the computation time of relative pose estimation to 40% of that of the ORB_SLAM3 front-end odometry, while ensuring that it is not too different from the ORB_SLAM3 front-end odometry in terms of accuracy and robustness. The effectiveness of this method was validated and analyzed using the EUROC dataset within the ORB_SLAM3 open-source framework. The experimental results serve as supporting evidence for the efficacy of the proposed approach.

## 1. Introduction

Using image frame information obtained from a camera to derive pose estimates, commonly known as odometry, has been a key research topic in the field of Simultaneous Localization and Mapping (SLAM) [1]. SLAM technology refers to the device carrying a sensor in an unknown environmental map with no prior environmental information through its own movement process to build the environment map and position the sensor in the map and focus on the real-time state of the sensor [2]. In various sensor types, cameras have the advantage of a lower cost and providing abundant environmental information, which makes them well-suited for subsequent tasks, such as identification, seg-mentation, and other semantic-based work. Hence, visual methods have emerged as a pivotal branch and a prominent research focus within the field of SLAM.

At present, visual odometry, typically serving as the front-end component of visual SLAM, is witnessing increasing adoption in more accessible mobile devices, like smartphones. This allows for the integration of more practical functionalities, thereby placing a greater emphasis on the real-time performance and lightweight operation of odometry algorithms. The core technical challenge is to achieve better accuracy and robustness with limited hardware computing resources. The odometry process known as bundle adjustment is employed to establish the correspondence between the two-dimensional pixel points in the image frames captured by the camera and the corresponding three-dimensional map points [3]. Many solvers exist for bundle adjustment, which efficiently solves the nonlinear least squares problem [4,5,6,7]. However, the fundamental challenge lies in establishing the correspondence between information from different frames, specifically relating to the same environmental features across different images. Currently, the classical approaches for addressing this challenge are optical flow and feature point methods. These two methods differ significantly in terms of their operating mechanisms, processing speed, and computational accuracy.

Optical flow is based on the variations in image brightness to estimate pixel-level motion. It assumes that the pixel intensities remain constant between adjacent frames during motion. Based on this assumption, optical flow calculates the motion vector for each pixel by tracking the changes in brightness across the image. On the other hand, the feature point method relies on extracting a key point from the image for motion estimation. These feature points typically possess unique positional and descriptor information, which enables them to exhibit good matching properties across different frames.

The feature point method generally incurs higher computational complexity com-pared to optical flow. This is primarily due to the intricate processes involved, such as feature extraction, feature matching, and feature tracking. Firstly, feature point extraction requires processing and calculations across the entire image. Secondly, the extracted feature points need to be matched with corresponding points in other frames, involving distance or similarity computations between features. Lastly, the feature point method estimates camera motion by tracking the movement of feature points across consecutive frames, necessitating matching and association operations. In contrast, optical flow operates at the pixel level, eliminating the need for feature extraction, matching, and tracking, thus resulting in a lower computational burden.

Compared to optical flow, the feature point method generally exhibits higher precision and robustness under normal circumstances. By extracting feature points and performing feature matching, the feature point method can provide more accurate camera motion estimation. Feature point possess unique positional and descriptor information, rendering them highly distinguishable and resilient during the matching process. On the other hand, optical flow operates at the pixel level, allowing for the estimation of motion vectors for each individual pixel. In cases of smooth motion and favorable lighting conditions, optical flow can deliver reasonably accurate results. However, optical flow is prone to failure in scenarios involving occlusions, texture deficiencies, or rapid motion, which may result in imprecise estimation outcomes.

Both optical flow and feature point methods possess unique advantages, and the fusion of these approaches has been a prominent area of academic research. One approach is to initially employ optical flow for rapid and coarse pose estimation between images, facilitating quick matching [8,9]. Subsequently, a subset of regions within the optical flow key point, or regions with high matching scores in optical flow, can be selected for feature point matching between the two images. This strategy effectively reduces the computational burden and time required for matching. However, this method faces the challenge of incorrect associations if the subset of regions selected based on coarse optical flow matching fails to establish accurate correspondences. For instance, if a matched optical flow key point lacks correct associations, such as a key point from the left side of image A being matched with key points from the right side of image B, the overall front-end matching will fail. Another approach involves incorporating an additional IMU (Inertial Measurement Unit) sensor. In this method, coarse matching still relies on optical flow, but the validity of matches is assessed by comparing the disparity between the estimated pose derived from image motion within the matching period and the integrated pose from the IMU. Feature point matching is subsequently performed based on this evaluation. However, this method necessitates sensor augmentation and improvements to the front-end algorithm, making it a hybrid solution that extends beyond pure visual odometry. Therefore, it is of great significance in the field of visual SLAM to develop visual odometry methods that strike a balance between accuracy, computational speed, and robustness while relying solely on visual information.

Motivated by this, this paper proposes a robust and integrated visual odometry framework exploiting optical flow and feature point methods that leverages optical flow tracking to accelerate the feature point matching process and obtain better-matched feature points by utilizing high-quality feature point selection. This will allow for accurate pose estimation using fewer feature points. When the performance of optical flow matching is poor, the odometry system switches to a global feature point method with a filtering mechanism to ensure both accuracy and robustness. By employing this approach, the system aims to mitigate the limitations of optical flow and improve the overall performance of the visual odometry.

The speed and accuracy of our odometry are experimentally verified and compared with the front-end odometry of the classical ORB_SLAM3 solution. A robust and integrated visual odometry framework exploiting the optical flow and feature point method exhibits more than double the speed of the front-end visual odometry in ORB_SLAM3. Despite the increased speed, the method maintains a similar level of measurement accuracy and robustness as the front-end visual odometry in ORB_SLAM3, which is based on the classical feature point method framework.

## 2. Related Work

Currently, the main methods for recovering camera poses and scene structures can be categorized into direct and feature point methods. For feature point-based visual odometry, the standard approach to solve this problem involves extracting a set of prominent image features in each image, using feature descriptors for continuous frame matching, and recovering camera motion and structure stably using pairwise polar geometry. Finally, poses and structures are optimized by minimizing the reprojection error. 

Most visual SLAM algorithms follow the basic idea presented in the literature [10], which has a good robustness of feature detectors and descriptors, enabling a good image frame matching in the presence of significant changes in illumination and angle. The MonoSLAM [2] system was the first real-time single-view SLAM system and has a milestone significance in the history of SLAM development. The PTAM [11] was the first SLAM scheme to use nonlinear optimization as a back-end; it introduces the key looping mechanism and also creatively realizes the parallelization of real-time localization and mapping processes, the first time in the history of visual SLAM to distinguish the concept of front-end and back-end. ORB_SLAM2 is the most typical characteristic-based SLAM system [12], and in 2020, Campos and others introduced ORB_SLAM3 [13] by improving ORB_SLAM2.

However, visual SLAM systems based on feature point methods have some drawbacks, including the need to handle robust estimate techniques corresponding to errors due to slow feature extraction and matching per frame [14,15]. Furthermore, most feature detectors prioritize accuracy over speed. Additionally, relying solely on well-localized, locally obvious features only exploits a small fraction of the image information available.

The direct method-based visual SLAM system estimates structure and motion directly by minimizing the error in the pixel-level intensity of the image [16]. It utilizes the magnitude and direction of the local intensity gradient in optimization, which is different from the feature point-based visual SLAM system that only considers feature location distance. The pixel correspondence is directly provided by the solution results, which eliminates the need for robust data correlation techniques. The direct method can also be divided into dense direct method and sparse direct method. DVO [17] and LSD-SLAM [18] are classic SLAM schemes based on the dense direct method. DSO [19] is a classic SLAM scheme based on the sparse direct method. However, this method requires a good initialization and therefore must be located in a favorable position of the cost function.

Due to the extreme complementarity between the advantages and disadvantages of the feature point method and the direct method, there are some schemes that combine the strengths of both methods. For example, [20] improved the traditional direct method by extracting features only for selected key frames, significantly reducing the computation time. After feature extraction, the direct method can quickly track features between two frames and has good local corner features that can track any pixel with non-zero intensity gradients.

The proposed robust and integrated visual odometry framework exploiting the optical flow and feature point method utilizes optical flow tracking to accelerate the traditional feature point method, improving the speed of odometry pose optimization while still retaining the global feature point method odometry to ensure robustness in the case of poor optical flow tracking.

## 3. Methods

The proposed odometry method utilizes optical flow and feature fusion to collect observation information from adjacent image frames. Initially, the object’s pose is estimated using optical flow, and key points are extracted from the image frames. Then, optical flow tracking is utilized to establish the matching relationship between neighboring frames, and to obtain a rough position estimation. Meanwhile, the matching relationship between neighboring frames established using optical flow tracking will also be used as the initial matching relationship for the subsequent local feature point method odometry. Tracking quantity judgment is introduced to evaluate the effectiveness of the optical flow matching results, based on the number of successfully matched key points.

**Figure 1:**
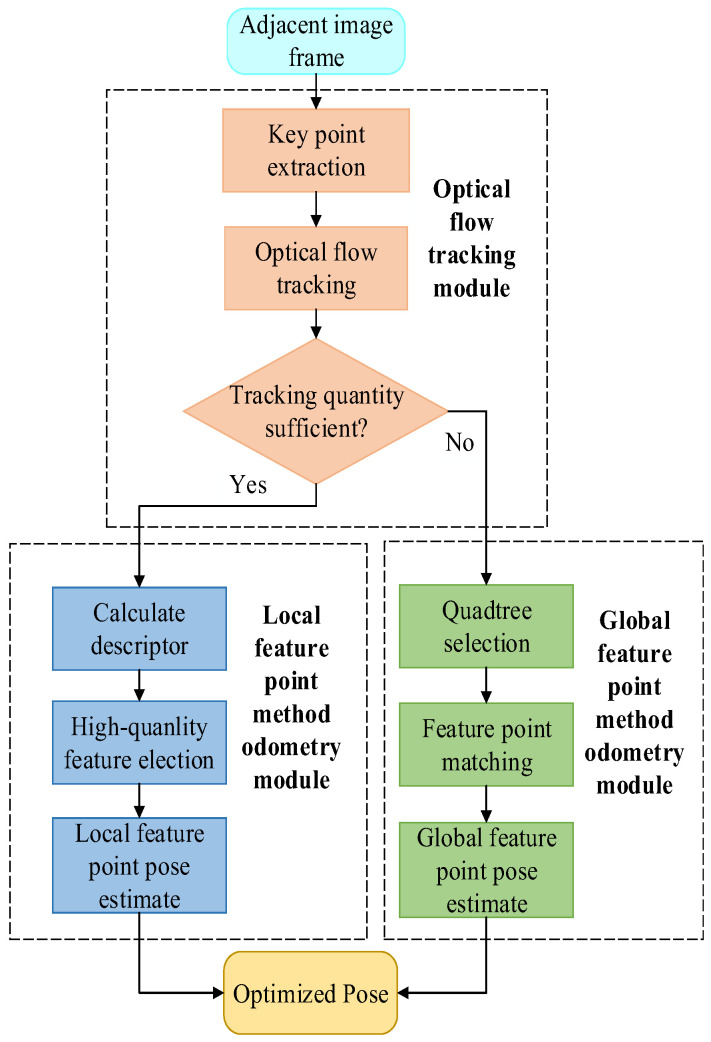
Framework of the robust and integrated visual odometry framework exploiting optical flow and feature point methods.

If the tracking quantity judgment determines that the number of key points is sufficient, the system proceeds to the local feature point odometry module. In this module, key point descriptors are computed, and high-quality feature points are selected for pose estimation using geometric constraints. On the other hand, if the number of successfully matched key points in the optical flow is too small, the odometry system switches to the global feature point odometry module. In this module, uniform distribution key point quadtree selection is performed to achieve an even distribution of key points. Subsequently, feature point matching is conducted, followed by pose estimation using geometric constraints to obtain an optimized pose. The specific data-processing flow is illustrated in Figure 1. The subsequent section provides a detailed introduction to the functionality of each module.

### 3.1. Optical Flow Tracking Module

The object collects its own observation information in the form of image frames into the optical flow tracking module; it first extracts all the key points in the image frames, and then performs optical flow tracking matching on the key points in adjacent image frames to obtain the key point optical flow tracking matching relationship between adjacent image frames.

#### 3.1.1. Key Point Extraction

The object feeds its own observation information into the optical flow tracking module and initially extracts all the key points from the image. The optical flow tracking module employs an improved version of the FAST key point detection method, which incorporates pre-detection based on FAST detection [21]. Common detection methods include FAST-9 and FAST-12. In the FAST-9 key point detection method, it is required that 9 consecutive pixels exceed the contrast threshold, while in the FAST-12 key point detection method, 12 consecutive pixels need to exceed the contrast threshold. For example, if A is the currently recognized key point, and B is one of the 16 pixels near A, then if the pixel gray of B is greater than 120% of the pixel gray of A or less than 80% of the pixel gray of A, it is considered that B exceeds the contrast threshold of A. However, traditional FAST-9 and FAST-12 also have some problems: FAST-9 only requires 9 consecutive pixels to exceed the detection threshold, thus leading to too many key points passing through the detection threshold and increasing the amount of calculation in subsequent steps. FAST-12 requires 12 consecutive pixels to exceed the threshold detection, which will cause some excellent pixels to fail the detection, such as 10 or 11 consecutive pixels exceeding the detection threshold. 

**Figure 2:**
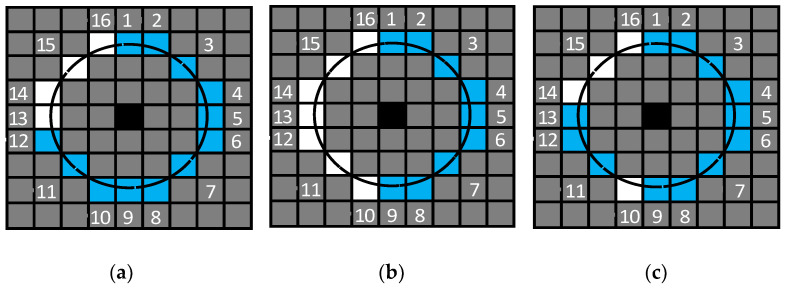
Threshold detection states of 16 pixels adjacent to pixels that are considered key points in different FAST key point detection methods. (**a**) Threshold detection of the FAST-12 key point detection method. (**b**) Threshold detection of the FAST-9 key point detection method. (**c**) Threshold detection of the key point detection method in our proposed odometry.

Therefore, the detection method in our proposed odometry takes into account the advantages of the above two methods. This method requires 9 consecutive pixels and a total of more than 12 pixels need to exceed the detection threshold, which requires both to ensure that most of the better pixels pass the threshold detection (9 consecutive points) and to ensure that the key points that pass threshold detection are good (a total of 12 points). Figure 2 shows the threshold detection states of 16 pixels adjacent to pixels that are considered key points in different FAST key point detection methods. Black pixels represent the key points currently recognized, while blue pixels represent pixels that exceed the detection threshold of the 16 pixels compared during detection; white pixels represent pixels that did not exceed the detection threshold among the 16 pixels compared during detection, and gray pixels represent other pixels near the currently evaluated pixel. From Figure 2, it can be intuitively seen that the requirements of FAST-9 are too lenient, and the requirements of FAST-12 are too strict, while the detection method in our proposed odometry takes into account the advantages of both methods, and its requirements are demonstrated very appropriately in future results.

The detection method in our proposed odometry still retains the pre-detection part (pick four pixels at locations 4, 8, 12, and 16 for threshold detection) of the FAST-12 key point detection method. Although a small number of pixels that meet the requirements cannot pass the pre-detection, the pre-detection can identify most of the pixels that do not meet the requirements in advance, which greatly improves the efficiency of key point detection. And the number of pixels that meet the requirements, but do not pass pre-detection, is a low proportion of all pixels that meet the requirements; so, the impact on critical point detection is quite limited.

**Figure 3:**
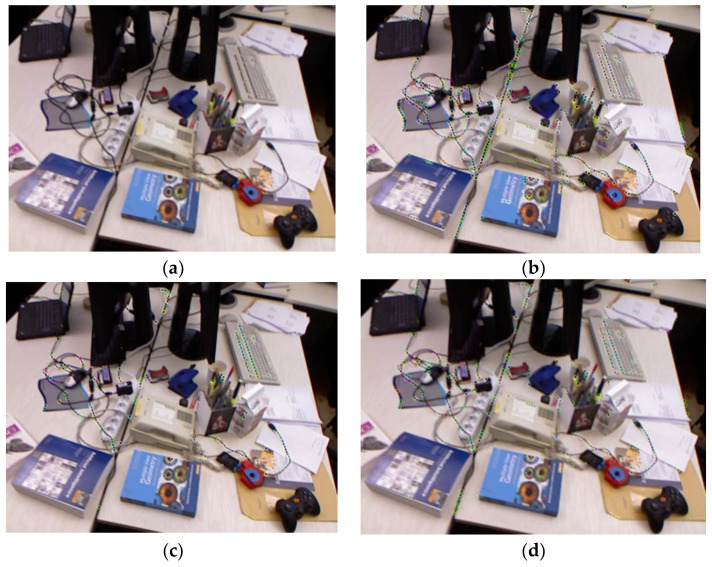
Comparison of the actual results of different FAST key point extraction methods. (**a**) Original image frame without key point extraction. (**b**) Image after extraction using the FAST-9 key point extraction method. (**c**) Image after extraction using the FAST-12 key point extraction method. (**d**) Image after extraction using the key point detection method in our proposed odometry.

In order to test the actual operation effect of different FAST key point extraction methods, we selected a colorful image frame from the rgbd_dataset_freiburg1_desk series of the TUM dataset, so as to obtain more key points and then better analyze the difference between the extracted key point detection method in our proposed odometry and those of the traditional FAST-9 and FAST-12. Through the actual test, we obtained the following data: FAST-9 key point detection method extracts 1032 feature points, FAST-12 key point detection method extracts 332 feature points, and key point detection method in our proposed odometry extracts 587 feature points. As it can be seen in Figure 3, the number of feature points obtained by the FAST-9 key point extraction method is too high, and more neighboring key points are extracted in some regions where the features are more obvious, which results in most of the key points being redundant for expressing the features of the whole image frame. Most of the key points obtained by the FAST-12 key point extraction method are located in the more obvious regions, and almost no key points are extracted in the edge regions of the image frame, which results in an uneven distribution of key points in the whole image frame. The key point detection method in our proposed odometry combines the advantages of the above two methods, so that the number of extracted key points is not too large, but also ensures that the distribution of key points in the whole image frame is more uniform.

#### 3.1.2. Optical Flow Tracking

All key points are matched between adjacent frames using optical flow tracking after they are extracted from the image frames. In our odometry, the Lucas–Kanade optical flow method [22] is used, and the matching relationship between key points in two adjacent image frames can be obtained by solving the minimum photometric error in the optical flow tracking process. The resulting corresponding key point matches are then sent to the tracking quantity judgment to determine whether the number of key points successfully matched by optical flow tracking is sufficient.

**Figure 4:**
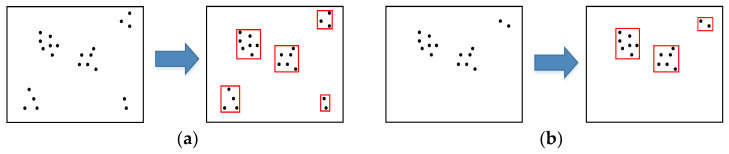
Image frame window divisions for different feature point detection methods. (**a**) Window division of our proposed feature point detection method. (**b**) Window division of the FAST-9 feature point detection method.

In the actual optical flow tracking process, our proposed odometry divides the neighboring key points in an image frame into multiple windows and assumes that the key points within each window have the same motion. The distribution of feature points is closely related to the number of windows. Taking part of the image frame in Figure 3 as an example, in Figure 4 we use black dots to represent the feature points and red squares to represent the delineated windows, using our key point extraction method, as in subplot (a) of Figure 4, the distribution of key points is more uniform and the number of windows is higher, while using the FAST-9 key point extraction method, as in subplot (b) of Figure 4, the key points are typically distributed non-uniformly and the number of windows is lower. When extracting the key points in an image frame, we have to make sure that the windows are distributed as evenly as possible over most of the image frame, so as to ensure that the feature information of the image frame is fully utilized.

The quality of the window division directly affects the quality of the optical flow tracking; if the window is too little and too dense, it may lead to the poor accuracy of the photometric error minimization results solved by the least squares method. If the window is too much and dense, it may lead to redundant feature information and excessive computation of photometric error minimization, thus affecting the efficiency of optical flow tracking. The key points extracted using the method proposed in the previous section are more uniformly distributed in the image frames, which is conducive to dividing the image frames into a more appropriate number of windows in the optical flow tracking process, thus ensuring the accuracy of the photometric error minimization solution and taking into account the efficiency of the optical flow tracking process.

### 3.2. Local Feature Point Method Odometry

If a sufficient number of key points are matched successfully by optical flow tracking, the input is then passed to the local feature point odometry module. First, the key point descriptors are calculated for the completed optical flow tracking, and then high-quality feature point selection is performed to extract only a few high-quality feature points from the image frames. Finally, the local feature point pose estimate is completed using geometric constraint relations to obtain the optimized pose.

#### 3.2.1. Computing Local Feature Point Descriptors

The matching relationship between key points is established through optical flow tracking in the optical flow tracking module, and the descriptors of successfully matched feature points are computed for the subsequent steps’ selection of high-quality feature points. In our proposed odometry, the BRIEF descriptor with rotational invariance is utilized. The optical flow method of our proposed odometry is not based on all pixels in the image frame but the key point after screening; so, usually, the key points for the success of optical flow tracking are usually two to three times that of the basis for this judgment, which greatly reduces the amount of calculation in the subsequent steps.

#### 3.2.2. High-Quality Feature Point Selection

After the computation of descriptors for the tracked key points, the selection of high-quality feature points was required. We used the score in Equation (1) to describe the quality of the feature points. The selection process considers the following three aspects: Firstly, the Hamming distance between the descriptors of two successfully tracked feature points in adjacent image frames, which is *H* in Equation (1). Secondly, the ratio of the Hamming distance between two successfully tracked feature point descriptors and the Hamming distance between each feature point and its neighboring feature point descriptors, which is *N* in Equation (1). Lastly, the absolute value of the angle between the optical flow tracing vector between two feature points and the average optical flow tracing vector between all successfully tracked feature points in adjacent image frames, which is *V* in Equation (1).

(1) $$SCORE=\frac{H_{\mathrm{best}}}{H_{i}}+\frac{N_{\mathrm{best}}}{N_{i}}+\frac{V_{\mathrm{best}}}{V_{i}}$$

The score in the equation is used to quantify the quality of the feature points, and the equation consists of three parts, *H*, *N*, and *V*. Each part is based on the metric of the best feature point in each image frame as the numerator, and the metric of the current feature point as the denominator, so that the larger the fraction, the better the score. The smaller Hamming distance indicates a higher likelihood that the two feature points correspond to the same spatial map point. The smaller ratio implies that the pair of feature points represents the correspondence between adjacent areas in a more representative manner. The smaller the absolute value of the angle, the better the tracking between two feature points in the entire image frame. By evaluating the feature points based on the above criteria, the best 20 feature points are selected among the successfully tracked optical flow points.

In the process of selecting high-quality feature points, we did not set specific quantitative indicators for the three evaluation indicators but calculated the percentage between all feature points and the best performing feature points based on the best performing feature points of each indicator. In this way, the disadvantage of poor adaptability of evaluation indicators caused by setting fixed quantitative standards was avoided. For example, in the same dataset, the Hamming distance between the feature points of the first two image frames is small, but the Hamming distance between the last two frames is large. If the indicator is set according to the first two frames, too many feature points in the last two frames will be deleted, which will affect the accuracy of subsequent pose estimation, and if the indicator is set according to the first two frames, too many feature points in the first two frames will be regarded as high-quality feature points, which will affect the calculation speed of subsequent pose estimation. 

Using relative indicators instead of absolute indicators can solve the problem of differences in indicators before and after in the same dataset. The quantitative indicator of the feature point with the best quality in an image frame was set to 1 (i.e., 100%), and the indicators of the other feature points were the percentages obtained by dividing them with the best indicators. The use of the relative percentages to indicate the indicator differences does not have the problem of non-uniformity of the units of the different indexes; so, there is no need to add a weight to the three indicators. The three indicators do not have the order of priority before and after, but according to the actual data obtained in the actual scene to judge the difference in importance between the indicators, without thinking that the priority of the indicators is set. Thus, the relative evaluation indicators have a good adaptability to different scenarios. When we select high-quality feature points, we only pay attention to the comprehensive indicator ranking of each feature point and do not pay attention to the specific performance of a feature point in an indicator.

#### 3.2.3. Local Feature Point Pose Estimate

Local feature point pose estimation was performed with the selected 20 high-quality feature points, and pose estimation was obtained by minimizing the reprojection error, which is based on the correspondence between the two-dimensional pixels of the image and the three-dimensional spatial coordinate points of the selected high-quality feature points. Since there are only twenty feature points for pose estimation, the calculation speed of the process is fast. Although the estimated number of feature points is small, these feature points are screened out after many comparisons; so, we believe that the pose correspondence between these local feature points can represent the pose correspondence between two image frames.

### 3.3. Global Feature Point Method Odometry

If the number of feature points successfully matched by optical flow tracking is too small, the global feature point odometry module is then established. In this module, uniform distribution feature point quadtree selection is first performed to achieve a more uniform distribution of feature points in the image frame. Subsequently, feature point matching is conducted, and finally, the optimized pose is obtained by applying geometric constraint relations to complete the global feature point pose estimate.

#### 3.3.1. Quadtree Selection

The distribution of key points in an image is often random, with key points being typically concentrated in specific areas. Computing key point descriptors for all key points in a small, dense area can be time-consuming and inefficient. In order to enhance the computational efficiency of global feature point descriptors, a method is proposed in our odometry that utilizes an improved quadtree with uniform distribution properties to select representative feature points in an image.

**Figure 5:**
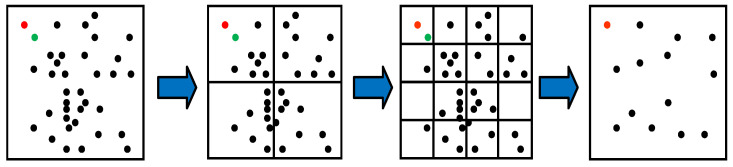
The process of improved quadtree feature point selection with uniform distribution properties.

The process of improved quadtree feature point selection with uniform distribution properties is depicted in Figure 5. The most representative feature point of the quadtree in each block is selected, according to the number of pixels of the continuous pass threshold detection in the 16 pixels near the key point in Section 3.1.1. Our method builds on this selection criterion and also considers the uniformity of the distribution of the selected key points in the image frame. Our method builds on this selection criterion and also considers the uniformity of the distribution of the selected key points in the image frame. In the case of an equal number of pixels of continuous pass threshold detection, priority is ascribed to the key points closer to the center of the block. For example, for the two feature points in the upper left corner of Figure 5, we labeled them as red and green points for easy differentiation, when the number of pixels of the continuous pass threshold detection of the two points is equal, the red point is selected as the representative point of the block, because the red point is closer to the center of the block than the green point.

As the global feature point odometry method requires computing descriptors for all feature points in the image frame, it consumes significant computational resources. In our odometry method, the quadtree feature point selection method is improved to enhance the uniform distribution property of the quadtree feature point selection method, which can greatly reduce the number of key points selected as feature points, reduce the amount of descriptor computation required, and make the distribution of feature points have better uniformity.

#### 3.3.2. Feature Point Matching

**Figure 6:**
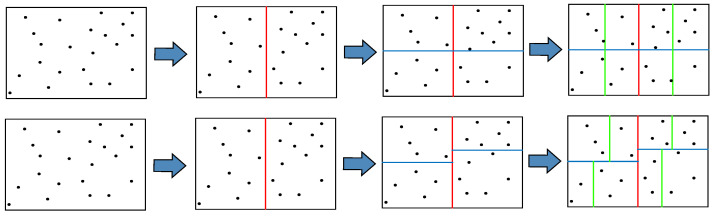
Comparison of the traditional k-d tree-building process and the improved k-d tree-building process in our odometry. (**top**) The traditional method of building a k-d tree for image frame feature points. (**bottom**) Our method of building a k-d tree for image frame feature points.

The feature point matching in the global feature point odometry method involves computing descriptors for the feature points selected through quadtree selection and establishing the matching relationship between feature points in adjacent image frames based on the Hamming distance between their descriptors. In our odometry, the nearest neighbor matching method [23] of the improved k-d tree is used to perform the global feature point odometry method. Figure 6 illustrates the comparison of the traditional k-d tree-building process and the improved k-d tree-building process in our odometry. Three bifurcations are represented by red, blue, and green segments. As it can be seen from Figure 6, the traditional k-d tree takes the average of the horizontal and ordinate coordinates of the image frame as the division basis, while our method takes the average of the horizontal and vertical coordinates of all key points in the image frame as the division basis.

In the feature point nearest-neighbor matching algorithm, feature points only need to match feature points in the same location area and adjacent area in the next image frame, but do not need to match all feature points in the next image frame. Using our method for region division, feature points in the image frame can be divided as evenly as possible, thereby improving the efficiency of feature point matching. Too many or too few feature points in a certain area can be avoided, too many feature points in one area lead to too much matching calculation, and too few feature points in one area lead to an insufficient matching accuracy.

#### 3.3.3. Global Feature Point Pose Estimate

The global feature point pose estimation follows a similar principle to local feature point pose estimation, which involves minimizing the reprojection error. However, there are differences between the two methods. In the global feature point pose estimation, the number of feature points considered is much larger than in the local feature point method, resulting in a longer computation time for the pose estimation process. It is important to note that the majority of the pose estimation work in this odometry system is accomplished through the local feature point method. The global feature point method is utilized only when optical flow tracking is not effective enough. Consequently, the global feature point method has a limited impact on the velocity of the system. However, it plays a crucial role in enhancing the robustness of the odometry system, despite its potential drawback of slower computation speed.

## 4. Experiments

The proposed robust and integrated visual odometry framework exploiting the optical flow and feature point method was implemented in C++ under Linux. Performance testing experiments were conducted on our proposed contribution using the MH05 (Machine Hall 05) sequence from the publicly available EUROC dataset. This dataset provides ground-truth location data obtained from the Leicra Total Station. The EuRoC dataset consists of video sequences captured using an AscTec FireFly UAV and flown repeatedly in an industrial environment, using a forward-looking camera [24].

The performance evaluation of our method involved conducting experiments to generate trajectories and assess the error between these trajectories and the ground truth values. TUM’s data format was applied, and trajectory evaluation was performed using EVO, a tool commonly used in the SLAM field for error evaluation. In order to achieve a fair and rational comparison, the speed, accuracy, and robustness of our robust visual odometry method, a comparison was made between our method and the front-end odometry part of the ORB_SLAM3 open-source framework. By conducting this comparison, the effectiveness of our method can be assessed, while ensuring fairness in the evaluation process, considering that our system primarily focuses on odometry.

Table 1 shows the Absolute Pose Error (APE) and Relative Pose Error (RPE) of the front-end visual odometry of ORB_SLAM3 and our proposed method. The absolute pose error refers to the root-mean-square error of each pose Lie algebra, as shown in Equation (2), to express the pose error between the actual measured trajectory and the truth trajectory.

(2) $$APE=\sqrt{\frac{1}{N}\sum_{i=1}^{N}{\left\|\log{\left(T_{gt,i}^{-1}T_{esti,i}\right)}^{\vee }\right\|}_{2}^{2}}$$

The relative pose error refers to each root-mean-square error that takes into account 1 to 2 moments, as shown in Equation (3). It also indicates the pose error method between the actual measurement trajectory and the real trajectory.

(3) $$RPE=\sqrt{\frac{1}{N-\Delta t}\sum_{i=1}^{N-\Delta t}{\left\|\log{\left({\left(T_{gt,i}^{-1}T_{gt,i+\Delta t}\right)}^{-1}\left(T_{esti,i}^{-1}T_{esti,i+\Delta t}\right)\right)}^{\vee }\right\|}_{2}^{2}}$$

From the table data, it can be observed that our proposed method achieves superior performance in multiple metrics for APE, except for the minimum value, where our method (0.0055) is slightly inferior to ORB_SLAM3. In terms of mean, median, min, root-mean-square error (RMSE), and standard deviation (STD), our method outperforms ORB_SLAM3. Even the sum of squared errors (SSE) and ORB_SLAM3 improved by 73.97%. This is because our method utilizes optical flow for initial filtering and uses multiple indicators to comprehensively screen feature points in the selection of high-quality feature points, resulting in higher quality local feature points compared to global feature points, thereby yielding better statistical performance in terms of matching error. Regarding RPE, our proposed method generally performs worse than ORB_SLAM3. STD and max are slightly below ORB_SLAM3; error scales are 100% worse than ORB_SLAM3 for min and median; and mean, RMSE, and SSE are approximately 50% worse. This is because RPE compares the performance differences between different instances of odometry. Our method uses a smaller number of feature points for matching, which introduces greater fluctuations in matching accuracy compared to global feature point matching, leading to a decreased stability in odometry.

**Figure 7:**
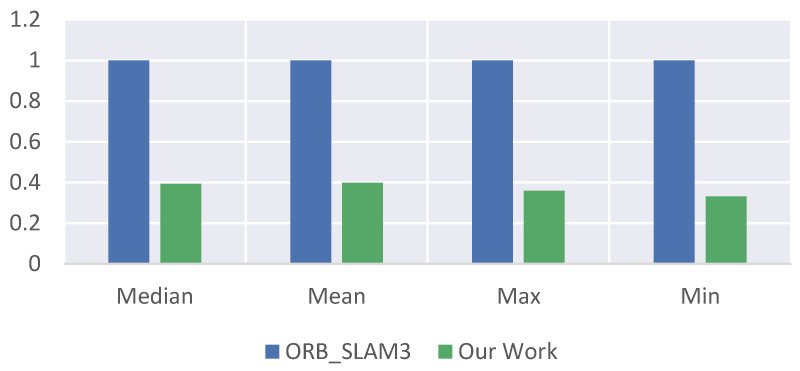
Comparison of the pose optimization speed of the front-end visual odometry of ORB_SLAM3 and our proposed odometry.

Table 2 shows a comparison of the computational time between the two methods, which is also the main advantage of our proposed method. It can be observed that, in terms of computation time, both the median time and mean time of our method are only about one-third of those of ORB_SLAM3. This improvement is significant and crucial for SLAM systems that require real-time performance. Also, since the absolute values between the different times are not consistent enough, we used the front-end odometry of the reference object ORB_SLAM3 as the unit one in Figure 7 and using the relative percentages to compare the times between the two methods is more convincing and makes the comparison more obvious.

**Figure 8:**
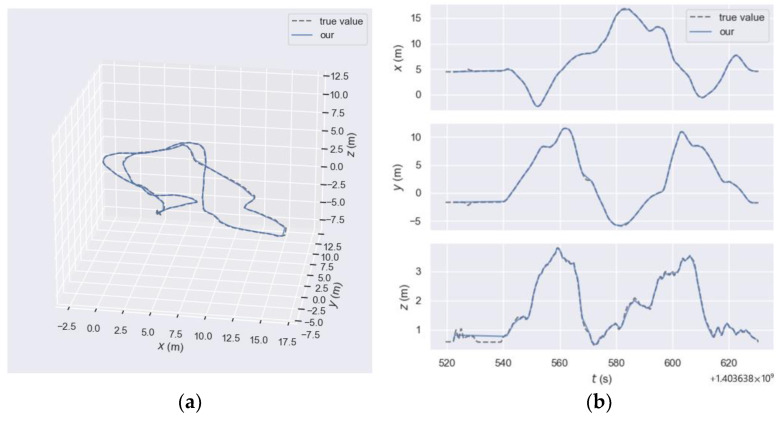
Comparison of the trajectory estimation and true value of our proposed odometry. (**a**) Comparison by actual three-dimensional motion trajectories. (**b**) Comparison of the *x*-axis, *y*-axis, and *z*-axis components of the motion trajectory separately.

In order to provide a more visual and intuitive comparison, the authors processed the data using the EVO tool, which allows for a better visualization of the numerical results, as shown in Figure 8. The dotted line represents the ground truth trajectory, and the solid line represents the trajectory of proposed method. As it can be seen from the subplot (a) of Figure 8, the trajectory of the robust and integrated visual odometry framework exploiting optical flow and feature point methods and the true value trajectory have a high degree of coincidence, indicating that the system has a good accuracy and robustness. It can be seen from subplot (b) of Figure 8 that the trajectory of the proposed method has a high consistency with the ground truth, and in the three-dimensional directions of space, except for the *z*-axis direction fluctuating at a few moments, the trajectory is more consistent in the *x*-axis and *y*-axis directions.

**Figure 9:**
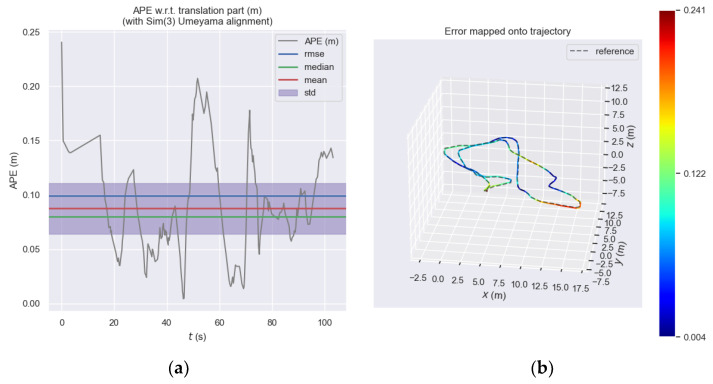
Absolute pose error of the front-end visual odometry of ORB_SLAM3. (**a**) Absolute pose error of the ORB_SLAM3 front-end visual odometry at each moment. (**b**) Absolute pose error of the ORB_SLAM3 front-end visual odometry represented by the color of the three-dimensional motion trajectory.

**Figure 10:**
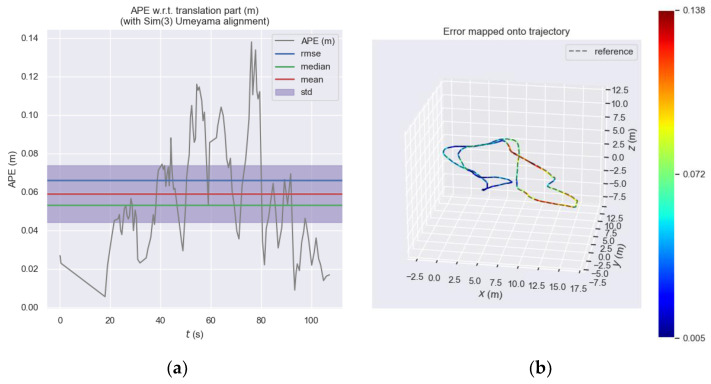
Absolute pose error of our proposed odometry. (**a**) Absolute pose error of our proposed odometry at each moment. (**b**) Absolute pose error of our proposed odometry represented by the color of the three-dimensional motion trajectory.

Figure 9 shows the absolute pose error of the front-end visual odometry of ORB_SLAM3 at each moment and Figure 10 shows the absolute pose error of our proposed odometry at each moment. Subplot (a) shows the error trend at each moment by the *y*-axis value and it be can seen that the error of the ORB_SLAM3 approximately ranges from 0 to 0.25, while the error of our proposed odometry approximately ranges from 0 to 0.14. Subplot (b) more intuitively expresses the different errors at each moment through the colors in the motion trajectory. The closer the color of the trajectory to zero, the greater the error, and the closer the color of the trajectory, the smaller the error. It should be noted here that the color is determined according to the proportion of its own error, and the same color in different figures does not mean that the error is the same but needs to be analyzed according to the color column on the right side of the figure. 

**Figure 11:**
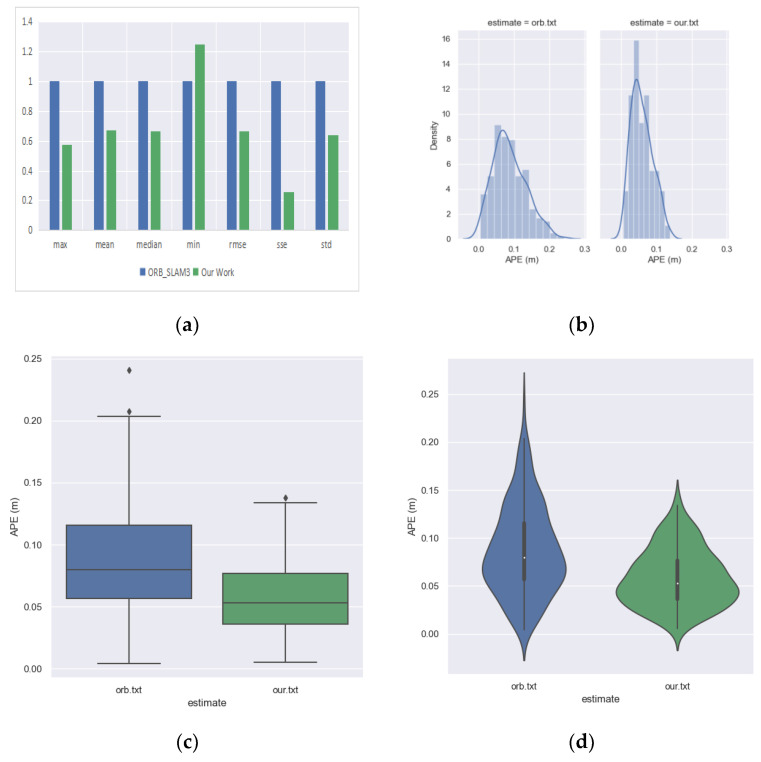
Comparison of the absolute pose error of the front-end visual odometry of ORB_SLAM3 and our proposed odometry. (**a**) The error comparison of ORB_SLAM3 and our odometry by histogram. (**b**) The error comparison of ORB_SLAM3 and our odometry by probability density plot. (**c**) The error comparison between ORB_SLAM3 and our odometry by box-line plot. (**d**) The error comparison between ORB_SLAM3 and our odometry by violin plot.

Some visualizations are used in Figure 11 to compare the absolute pose error of the ORB_SLAM3 and odometry. In the subplot (a) of Figure 11, it is not appropriate to compare the absolute values of different indicators (especially the max and min) because they vary greatly; if placed on the same coordinate system, the contrast differences in ‘min’ will not be well demonstrated. Therefore, we used the indicator of the ORB_SLAM3 as unit 1 and compared our methods in the form of relative values, which allows for a more intuitive and clearer view of the differences between the two methods. From the subplot, it can be visualized that our odometry outperforms ORB_SLAM3 on most error indicators. The subplot (b) of Figure 11 shows that the mean and variance of the absolute errors for our odometry is smaller than those for ORB_SLAM3, the diamond symbols above the box shapes in subplot (c) of Figure 11 indicate outliers, so it can be seen that both methods have fewer outliers, and the subplot (d) of Figure 11 shows that our odometry has a more concentrated distribution than ORB_SLAM3. This is due to the fact that our odometry uses high-quality feature point selection that eliminates most of the critical points; fewer feature points are selected but their metrics perform better, and thus our odometry has smaller absolute error means and variances and a more concentrated distribution of feature points.

**Figure 12:**
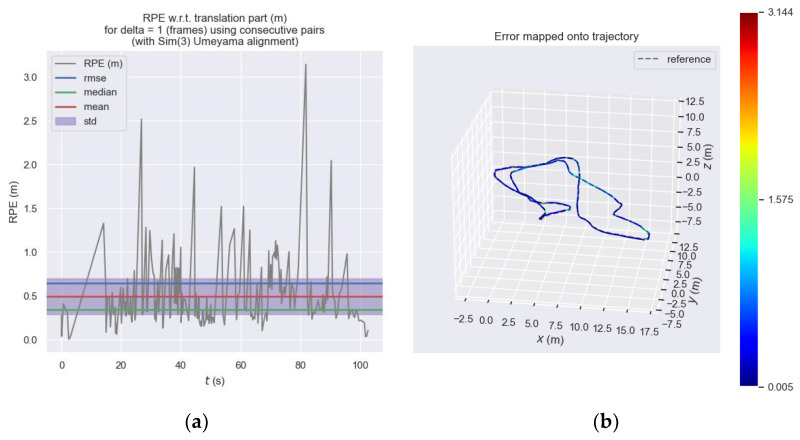
Relative pose error of the front-end visual odometry of ORB_SLAM3. (**a**) Relative pose error of the ORB_SLAM3 front-end visual odometry at each moment. (**b**) Relative pose error of the ORB_SLAM3 front-end visual odometry represented by the color of the three-dimensional motion trajectory.

**Figure 13:**
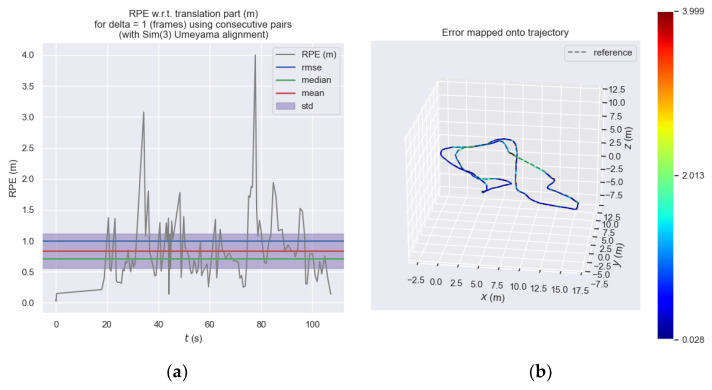
Relative pose error of our proposed odometry. (**a**) Relative pose error of our proposed odometry at each moment. (**b**) Relative pose error of our proposed odometry represented by the color of the three-dimensional motion trajectory.

Figure 12 shows the relative pose error of the front-end visual odometry of ORB_SLAM3 at each moment and Figure 13 shows the relative pose error of our proposed odometry at each moment. Subplot (a) expresses the trend of error at each moment on the *y*-axis and it can be seen that the error of the ORB_SLAM3 approximately ranges from 0 to 3.2, while the error of our proposed odometry approximately ranges from 0 to 4.0. We considered this performance difference to be acceptable and within the same order of magnitude. Subplot (b) is more intuitive to express the different errors at each moment through the color in the motion trajectory. 

**Figure 14:**
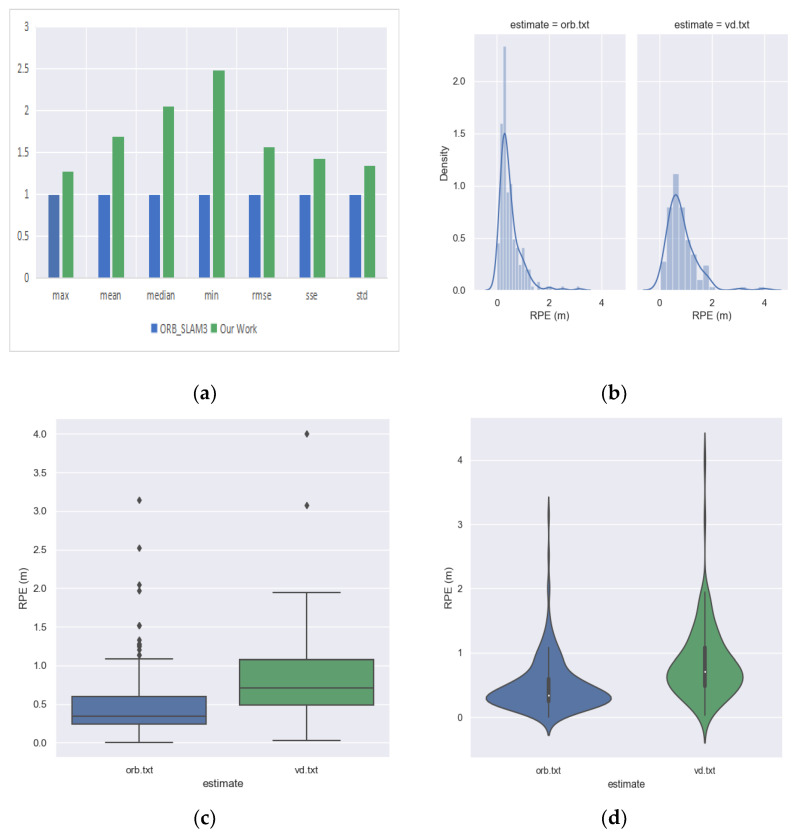
Comparison of the relative pose error of front-end visual odometry of ORB_SLAM3 and our proposed odometry. (**a**) The error comparison of ORB_SLAM3 and our odometry by histogram. (**b**) The error comparison of ORB_SLAM3 and our odometry by probability density plot. (**c**) The error comparison between ORB_SLAM3 and our odometry by box-line plot. (**d**) The error comparison between ORB_SLAM3 and our odometry by violin plot.

Some visualizations are used in Figure 14 to compare the relative pose errors of ORB_SLAM3 and our odometry. The subplot (a) of Figure 14 visually shows that the error metrics of our odometry is slightly inferior to that of ORB_SLAM3, but roughly in the same order of magnitude, the subplot (b) of Figure 14 shows that the mean and variance of the absolute errors of our odometry is larger than those of ORB_SLAM3, the diamond symbols above the box shapes in subplot (c) of Figure 14 indicate outliers, so it can be seen that both methods have fewer outliers and ORB_SLAM3 has more outliers, and the subplot (d) of Figure 14 shows that the distribution of our odometry is larger than ORB _SLAM3, which is more sparse.

From the above table and figure, it can be concluded that the robust and integrated visual odometry framework using the optical flow and feature point method is more than twice as fast than the front-end visual odometry of ORB_SLAM3 in terms of speediness. ORB_SLAM3’s front-end visual odometry has a median time of 0.0573, a mean time of 0.0667, a max time of 0.2011, and a min time of 0.0166; our odometry has a median time of 0.0225, a mean time of 0.0265, a max time of 0.0721, and a min time of 0.0055. In terms of accuracy, the absolute pose error of our method outperforms the front-end visual odometry of ORB_SLAM3, while the relative pose error is slightly inferior to that of ORB_SLAM3. This is because most of the image frames are estimated by local feature point odometry for posing estimation, and the process only needs to compute some local high-quality feature points instead of all of them; so, the position optimization of our odometry is significantly faster than that of the front-end visual odometry of ORB_SLAM3. The main role of the global feature point method odometry module is to ensure the robustness of the whole odometry.

**Figure 15:**
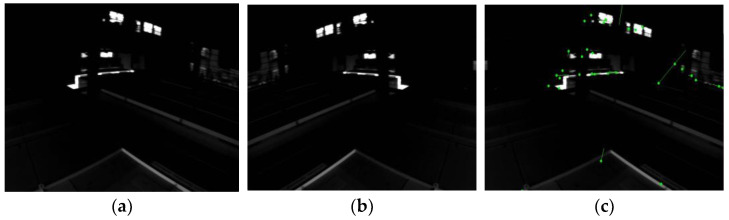
Optical flow tracking effect in low-light scenes. (**a**) An image frame from a low-light scene in the EUROC dataset. (**b**) The adjacent image frame in the low-light scene in the EUROC dataset. (**c**) The effect of two image frames for optical flow tracking in (**a**,**b**).

We also conducted robustness experiments on our odometry, through which we found that, if we do not include the global feature point method odometry module in the odometry, the robustness of this odometry becomes poorer in optical flow tracking. The robustness of the odometry is significantly improved by adding the global feature point odometry module to the odometry, and tracking failure almost never occurs. The reason for this is that, in some special scenes, such as the low-light scene in Figure 15, Subplot (c) in Figure 15 shows that the green dots indicate feature points that were successfully tracked by the optical flow, and the green line indicates the optical flow tracked by the feature points. it can be observed that the successfully tracked feature points are sparse, the consistency of the tracking direction of the feature points is very poor, and the direction of most feature point tracking trajectories differs significantly from the direction that can be judged by the naked eye. Although it is not common to have this kind of low-light situation, this problem is fatal for odometry, which emphasizes real-time performance. Therefore, this experiment fully demonstrates that the global feature point method odometry module plays a crucial role in the robustness of the overall odometry, which is one of the more important advantages of our odometry. Future work will focus on more diverse open-source datasets to validate the generalizability of this method and conduct testing on practical hardware systems.

## 5. Conclusions

In our study, the acceleration of the feature point matching process in visual odometry was achieved by optical flow tracking, which improves the calculation speed of robust and integrated visual odometry framework exploiting optical flow and feature point methods and retains the traditional global odometry to ensure the robustness of our odometry. We experimentally evaluated the rapidity, accuracy, and robustness of the front-end visual odometry of ORB_SLAM3 and the robust and integrated visual odometry framework exploiting optical flow and feature point method. Our odometry achieved a balance between speed, accuracy, and robustness for the following four reasons: (i) Since the core of the scheme is still the feature point-based method, the system can guarantee the accuracy. (ii) The scheme uses global feature point method of visual odometry when the number of optical flow tracking is not sufficient, and therefore improves the robustness of the whole system. (iii) The scheme performs the optical flow tracking of feature points in the image before the pose estimate by the local feature point method of visual odometry. It is equivalent to the initial pose estimation before the odometry pose estimate, which provides a good initial value for the subsequent local feature point method pose estimate and improves the computational efficiency of the pose estimate. (iv) This scheme uses optical flow tracking to accelerate the odometry process and does not require the computation of descriptors for all the feature points in the image frame as in the traditional feature point method of visual odometry, resulting in a significant increase in computational speed.

## Figures and Tables

**Table 1:** Comparison of absolute and relative pose errors of the front-end visual odometry of ORB_SLAM3 and our proposed odometry.

	ORB_SLAM3		Our Work		Improvements	
	APE	RPE	APE	RPE	APE	RPE
max	0.2405	3.1440	0.1379	3.9988	42.66%	−27.19%
mean	0.0874	0.4951	0.0589	0.8369	32.61%	−69.04%
median	0.0799	0.3449	0.0531	0.7093	33.54%	−105.65%
min	0.0044	0.0052	0.0055	0.0129	−25.00%	−148.08%
rmse	0.0991	0.6436	0.0659	1.0038	33.50%	−55.97%
sse	2.0701	86.9772	0.5388	123.9393	73.97%	−42.50%
std	0.0465	0.4111	0.0296	0.5542	36.34%	−34.81%

**Table 2:** Comparison of the pose optimization speed of the front-end visual odometry of ORB_SLAM3 and our proposed odometry.

(Seconds)	ORB_SLAM3	Our Work	Improvements
Median time	0.0573	0.0225	60.07%
Mean time	0.0667	0.0265	60.27%
Max time	0.2011	0.0721	64.15%
Min time	0.0166	0.0055	66.87%

## Data Availability

The data sources used in this article are from the publicly available EuRoC MAV Dataset provided by the Swiss Federal Institute of Technology in Zurich (ETH). Readers can find relevant information about the data and download links at https://projects.asl.ethz.ch/datasets/doku.php?id=kmavvisualinertialdatasets.

## References

[1] Liu H., Zhang G., Bao H. (2016). A survey of monocular simultaneous localization and mapping. J. Comput.-Aided Des. Comput. Graph..

[2] Davison A.J., Reid I.D., Molton N.D., Stasse O. (2007). MonoSLAM: Real-time single camera SLAM. IEEE Trans. Pattern Anal. Mach. Intell..

[3] Triggs B., McLauchlan P., Hartley R., Fitzgibbon A., Triggs W., Zisserman A., Szeliski R. (2000). Bundle adjustment—A modern synthesis. Vision Algorithms: Theory and Practice.

[4] Dellaert F., Kaess M. (2006). Square Root SAM: Simultaneous localization and mapping via square root information smoothing. Int. J. Robot. Res..

[5] Kaess M., Johannsson H., Roberts R., Ila V., Leonard J.J., Dellaert F. (2012). iSAM2: Incremental smoothing and mapping using the Bayes tree. Int. J. Robot. Res..

[6] Agarwal A., Mierle K., The Ceres Solver Team Ceres Solver. http://ceres-solver.org.

[7] Kummerle R., Grisetti G., Strasdat H., Konolige K., Burgard W. g2o: A general framework for graph optimization. Proceedings of the 2011 IEEE International Conference on Robotics and Automation.

[8] Bar-Shalom Y., Li X.R., Kirubarajan T. (2001). Estimation with Applications to Tracking and Navigation.

[9] Huang G.P., Mourikis A.I., Roumeliotis S.I. (2010). Observability-based rules for designing consistent EKF SLAM estimators. Int. J. Robot. Res..

[10] Scaramuzza D., Fraundorfer F. (2011). Visual odometry [tutorial]. Part I: The first 30 years and fundamentals. IEEE Robot. Autom. Mag..

[11] Klein G., Murray D. Parallel tracking and mapping for small ar workspaces. Proceedings of the 2007 6th IEEE and ACM International Symposium on Mixed and Augmented Reality.

[12] Mur-Artal R., Tardos J.D. (2016). ORB-SLAM2: An Open-Source SLAM System for Monocular, Stereo and RGB-D Cameras. IEEE Trans. Robot..

[13] Campos C., Elvira R., Rodríguez J.J.G., Montiel J.M., Tardós J.D. (2020). ORB-SLAM3: An Accurate Open-Source Library for Visual, Visual-Inertial and Multi-Map SLAM. IEEE Trans. Robot..

[14] Fischler M.A., Bolles R.C. (1981). Random sample consensus: A paradigm for model fitting with applications to image analysis and automated cartography. Commun. ACM.

[15] MacTavish K., Barfoot T.D. At all costs: A comparison of robust cost functions for camera correspondence outliers. Proceedings of the 2015 12th Conference on Computer and Robot Vision.

[16] Irani M., Anandan P. All about direct methods. Proceedings of the International Workshop on Vision Algorithms.

[17] Kerl C., Sturm J., Cremers D. Dense visual slam for rgb-d cameras. Proceedings of the 2013 IEEE/RSJ International Conference on Intelligent Robots and Systems.

[18] Engel J., Schops T., Cremers D. Lsd-slam: Large-scale direct monocular slam. Proceedings of the European Conference on Computer Vision.

[19] Gao X., Wang R., Demmel N., Cremers D. Ldso: Direct sparse odometry with loop closure. Proceedings of the International Conference on Intelligent Robots and Systems.

[20] Forster C., Zhang Z., Gassner M., Werlberger M., Scaramuzza D. (2017). SVO: Semidirect Visual Odometry for Monocular and Multicamera Systems. IEEE Trans. Robot..

[21] Rosten E. (2006). Machine learning for high-speed corner detection. Proceedings of the European Conference on Computer Vision.

[22] Lucas B.D., Kanade T. (1997). An Iterative Image Registration Technique with an Application to Stereo Vision. Proceedings of the 7th International Joint Conference on Artificial Intelligence.

[23] Muja M. Fast approximate nearest neighbors with automatic algorithm configuration. Proceedings of the VISSAPP.

[24] Burri M., Nikolic J., Gohl P., Schneider T., Rehder J., Omari S., Achtelik M.W., Siegwart R. (2016). The EuRoC micro aerial vehicle datasets. Int. J. Robot. Res..

